# Anatomical constitution of sense organs as a marker of mental disorders

**DOI:** 10.3389/fnbeh.2015.00059

**Published:** 2015-03-10

**Authors:** Francisco Güell, Javier Bernácer

**Affiliations:** Mind-Brain Group, Institute for Culture and Society, University of NavarraPamplona, Spain

**Keywords:** neurodevelopment, perception, schizophrenia, sensibility, vision

It is challenging to set the spatial limits of the mind. This was suggested in classical philosophy by Aristotle, when he stated that the mind is “in a way all existing things” (Aristotle, 1986) but it is also a trendy topic studied by the extended mind theorists, for example (Clark and Chalmers, 1998). The latter propose that the mind goes beyond the body in the act of cognition, since the environment plays a crucial role in mental activity. In this opinion article we do not go that far, but we pose an important question: where in the body shall we look for markers of mental disorders? Our proposal is that the bodily constitution of sense organs may be an indicator of mental characteristics, and therefore it can be affected in cases of disorders such as schizophrenia. We support this opinion with empirical papers and we suggest new lines of research to improve the understanding of this devastating disease.

Ever since Occidental thought began to flourish amongst the Greeks, sensibility and intelligence have been considered different faculties. They are intimately related, but have different objects. In general terms, lower faculties nourish higher capacities, with intelligence being the highest of them. This intuitive description is very much common sense: the sense organs are stimulated and transfer signals to the central nervous system through peripheral nerves. The faculty of sensibility lies in sense organs, and higher faculties are related to the brain, which is in charge of processing and elaborating a response. This way of explaining animal and human behavior is computational in the sense that we have an input, a process and an output. Taking the visual system as an example, the input is the stimulation of cones and rods by photons, which elicits an action potential; the process takes place in the lateral geniculate nucleus of the thalamus and the visual cortex, and the output is the perceived image. Nowadays we know that top-down signals are as important as bottom-up influences in perception, and it is widely accepted that anatomical constitution is an important factor to guarantee the accuracy of these signals. In the case of the bottom-up flow of information, the constitution of sense organs involves not only neural tissue (i.e., the retina), but also non-neural related structures (i.e., crystalline lens, eye muscles). Moreover, anatomical constitution plays an important role in perception and mental disorders (Korn, 2000; Eskelinen et al., 2013).

From a computational point of view, mental disorders such as schizophrenia are mainly considered an impairment at the level of the “process,” and therefore their physical correlates are searched for in the brain. Furthermore, errors in the process are expressed as a disturbance of the output, that is, behavior. Hence, the diagnosis of mental disorders is mostly carried out after observations, interviews, and the comparison of the results with diagnostic manuals. In any case, research on psychiatric conditions has focused on the search for brain correlates at the level of the process. For example, an aberrant migration of cortical interneurons during development has been found to be associated with schizophrenia.The migration of interneurons in the embryo is fundamental for correct mental function, as it determines the final positioning of the neurons, thereby establishing the basis for the correct wiring of neural circuitry (Marín, 2013). Multiple investigations suggest that schizophrenia and other neuropsychiatric disorders are associated with interneuron dysfunction (Marín, 2012), and that schizophrenia patients carry mutations in certain genes that affect the migration of the cortical interneurons during embryonic development (Valiente and Marín, 2010). Another clear example of a physical correlate is the fact that a progressive loss of brain volume occurs in schizophrenia, affecting both gray and white matter. Brain volume loss in schizophrenia seems to be related to a combination of early developmental processes (reflected in intracranial volume reduction) as well as the progression of the illness (Haijma et al., 2013).

There are a plethora of studies that support a physical modification of the brain in schizophrenia, which correlates with a disruption in the “computational process.” In addition, we have known for decades that perception also plays an important role in the understanding of schizophrenia and the detection of its symptoms (McGhie and Chapman, 1961). There are several studies that show a correlation between schizophrenia and the alteration of visual perception (such as visual masking, Green et al., 2012, luminance, size, contrast, orientation and motion perception, Yang et al., 2013; Serrano-Pedraza et al., 2014), as well as eye movements (Silverstein and Keane, 2011). However, vision is not the only sense affected in schizophrenia: aberrant processing of auditory (Micoulaud-Franchi et al., 2014), olfactory (Moberg et al., 2014), gustative (Compton et al., 2013) and tactile (Ferri et al., 2014) stimuli also occurs. These are important discoveries for improving our understanding of schizophrenia.

The main message of these introductory paragraphs is that in schizophrenia there are perceptual anomalies whose physical substrate has been searched for at the level of the process, that is, in the brain. We will suggest a possible new approach to diagnosing mental disorders such as schizophrenia, i.e., looking for physical differences directly in the constitution of sense organs rather than impairments in the processing of the signals coded by them. In order to simplify our arguments and make them as clear as possible, we will focus on the study of vision in schizophrenia.

Previous reports have shown an association between visual perception anomalies, as well as aberrant eye-related movements, and schizophrenia. An extensive review of the topic is beyond the scope of this article, so we will only mention some examples to show how this association has been studied in this disorder. A special section of the journal *Schizophrenia Bulletin*, published in 2011 and edited by Silverstein and Keane (2011), analyzes the contribution of vision science to this mental illness. According to the editors, this special section refers to “psychophysical, electrophysiological and imaging data,” focusing on intermediate vision—mainly object processing. The extensive research that begins the section describes several behavioral, neuroimaging and neurophysiological studies published between 2005 and 2010, all of which demonstrate perceptual organization impairment in schizophrenia. They point to an association between aberrant object processing and a compromised neural signal in visual extrastriate areas and the temporal, parietal and frontal regions related to the integration of visual information. This mainstream view is supported by a recent review on visual illusions and schizophrenia, interpreted via a Bayesian perspective (Notredame et al., 2014). In this case, perception is interpreted as the interplay between top-down expectations and bottom-up sensory inputs; therefore, the cortical influence on sensory signals is crucial in object processing. Visual illusions will emerge when expectations and bottom-up signals are abnormally integrated, as Dima et al. (2010) empirically demonstrate: in their experiment, healthy control subjects seem to experience visual illusions due to the strong influence of top-down expectations; on the other hand, schizophrenia patients overcome the illusions through a strengthened bottom-up input. With respect to eye-related movements, which are an indicator of the constitution of sense organs, a recent report shows that simple tests for the detection of abnormal eye movements can accurately discriminate between schizophrenia patients and controls (Benson et al., 2012). These researchers evaluated different oculomotor tasks and found differences in nearly all of them. Moreover, fixation dispersal during free viewing was the task that best discriminated between patients and controls. With respect to the etiology of this finding, the authors hypothesize that the eye movement disturbance should result from aberrant physiological processes directly connected to those which are responsible for the pathophysiology of schizophrenia.

The new perspective we propose here is based on the actual unity of sensibility and cognition or, in other words, the integrity of the input-process-output framework. In our opinion, mental diseases can be accompanied by physical anomalies in the sense organs; this should not be overly surprising if we consider that some disorders, such as schizophrenia, are even associated with alterations in non-neural peripheral tissues (Fernandez-Egea et al., 2009; Dieset et al., 2014). Our statement is based on two well-proven facts: (1) schizophrenia has been proven to have a genetic component (Schizophrenia Working Group of the Psychiatric Genomics Consortium, 2014); (2) the physical substrate of sense organs and neural tissue derive from the same embryonic layer, the ectoderm, which is in a continuous interplay with the mesoderm, where the non-neural structures of the sense organs originate from. In fact, going back to the case of vision, our theoretical hypothesis is already supported by several recent studies that suggest anatomical and functional anomalies of the retina in schizophrenia (Fountoulakis, 2010; Hébert et al., 2010; Chu et al., 2012; Lee et al., 2013; Lavoie et al., 2014). At a genetic level, there is also evidence that supports this novel approach. As we have noted in a recent article (Güell, 2014), four of the five known genes involved in the disrupted tangential migration of cortical interneurons (NRG1, ERBB4, GRIN1, and DTNBP1) are involved in the expression of molecules related to visual structures. We believe that it could be of great interest to assess how the expression of these genes (and their mutations) may impact the anatomical constitution of the visual system, since this feature could be used as a new diagnostic tool in schizophrenia. Indirectly, it may also be interesting to analyze the differences in eye morphology in controls and schizophrenia patients who carry mutations in any of these genes.

This hypothesis is supported by other experiments. The work by Benson and collaborators cited above (Benson et al., 2012) focuses on the physiological (functional) level of the visual system and we wonder whether the anomalies they found could be associated with a modification of eye morphology. In this sense, it could be interesting to enrich these functional studies with an analysis of the anatomical features of the eye and eye-related structures, such as retinal nerve fiber layer, macular volume, proprioceptive information of eye muscles (Korn, 2000; Chu et al., 2012; Lee et al., 2013), or using techniques such as adaptive optics (Saleh et al., 2014). Furthermore, Yang et al. (2013) suggest than anomalous perceptual experiences might lie at the base of the chronic difficulties confronted by individuals with schizophrenia, as they attempt to navigate the complex, fast-moving and constantly changing world. Going back to the causal chain, even though part of these perceptual anomalies might be due to top-down expectations from the cerebral cortex, it should be taken into account that subtle changes in the morphological features of the receptor organs could also contribute to these aberrant experiences. Since perception is a continuous interplay between top-down and bottom-up signals, we believe the study of the constitution of the sense organs could be as important as analyzing the anatomy and function of the brain.

We are not trying to identify perception and cognition in our proposal. Rather, we suggest that the diagnosis of a complex mental disorder could be done by looking for these morphological traits in the sense organs, as a complement of neuroimaging techniques. Therefore, we propose that research on schizophrenia and other mental disorders could profitably focus on the anatomical constitution of the input structures, as well as on brain correlates. Thus, the notion of “endophenotype” introduced by Gottesman and Shields (1973) as “internal phenotypes discoverable by a biochemical test or physiological marker” could be expanded to the morphological features of the peripheral nervous system and its associated structures. This could provide new tools to improve diagnosis, adequately understand the etiology of the disease, and eventually open new paths for the treatment of these disorders.

As a final note, we would like to clarify that this potential association between the morphology of input structures and mental disorders does not mean a radical determinism. Even though certain morphological configurations may be related with a disease, they would not necessarily imply that a person is “schizophrenic,” or even as being on the “schizophrenic spectrum.” Rather, it would point to a constitutional predisposition (Güell, 2014) to suffer the disease, open to various environmental factors.

## Conflict of interest statement

The authors declare that the research was conducted in the absence of any commercial or financial relationships that could be construed as a potential conflict of interest.
